# Cyclotherapy: opening a therapeutic window in cancer treatment

**DOI:** 10.18632/oncotarget.524

**Published:** 2012-06-16

**Authors:** Ingeborg M.M. van Leeuwen

**Affiliations:** ^1^ Microbiology, Tumor and Cell Biology, Karolinska Institutet, Stockholm, Sweden

**Keywords:** Cyclotherapy, chemotherapy, p53, nutlin-3, tenovin-6, leptomycin B, actinomycin D

## Abstract

Cyclotherapy is a promising endeavor to improve cancer treatment by tackling the dose-limiting side effects of chemotherapy, especially for cancers harboring mutations in the TP53 tumor suppressor. In this particular context, pre-treatment with a p53 activator halts proliferation in healthy tissue, while leaving the p53-deficient tumor susceptible to conventional chemotherapy.

Cyclotherapy is a promising endeavor to improve cancer treatment by tackling the dose-limiting side effects of chemotherapy. The origins of chemotherapy can be traced back to WWII, when it was accidentally discovered that nitrogen mustard transiently suppressed lymphoma. Over the decades that followed, the principle that highly toxic compounds can be used to combat cancer led to the identification and subsequent clinical approval of a series of “classic” chemotherapeutics [1, 2]. Early examples include the still widely used vincristine (*Oncovin*®) and dactinomycin (*Cosmegen*®). The majority of anticancer drugs that are on the market today belong to the same category as these; that is, they are cytotoxic compounds with cancer-nonspecific targets such as tubulin or DNA [3, 4]. The most vulnerable tissues are those with a high proliferative ratio, independent of whether they are normal or cancerous. Due to this lack of selectivity, side effects are prevalent and patients suffer from bone marrow suppression, neutropenia, anemia, nausea and vomiting, amongst other ailments. In addition, they are subjected to an increased risk of developing second tumors later in life.

Advances in the understanding of the molecular and cellular biology of cancer are making the identification of novel therapeutics targeting cancer- or tissue-specific traits possible. In-depth knowledge about the genetic alterations responsible for chronic myelogenous leukemia, for instance, enabled the rational design of imatinib mesylate (*Gleevec*®) [5]. In addition to the development of more selective anticancer agents, research efforts are also focused on improving the cure rate with existing drugs, for example, by optimizing drug combinations or refining drug delivery to a tumor. Another promising strategy aimed at increasing the therapeutic window of chemotherapy involves reducing the sensitivity of healthy tissue to anticancer agents. For cytotoxic drugs, which indiscriminately target cycling cells, chemoprotection can be achieved by selectively inducing a transient cell cycle arrest in normal cells. This concept, known as cyclotherapy [6, 7], is illustrated in Figure 1 for cancers harboring mutations in the *TP53* tumor suppressor. In this particular context, pre-treatment with a p53 activator halts proliferation in healthy tissue, while leaving the p53-deficient tumor susceptible to conventional chemotherapy [8-10].

**Figure 1:**
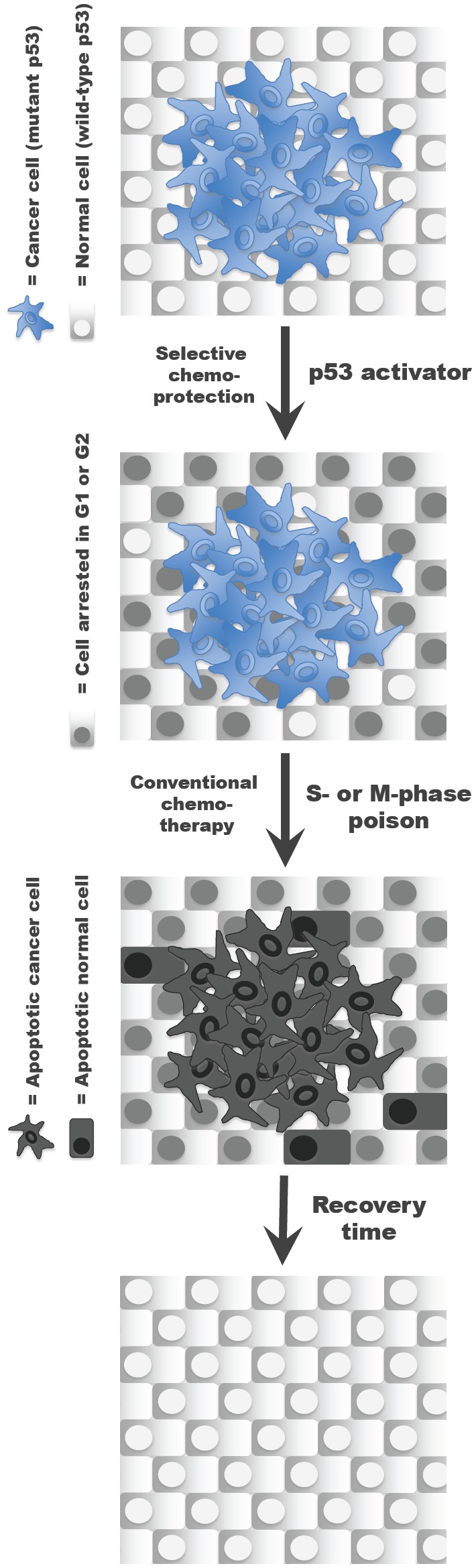
The cyclotherapy concept illustrated for patients with p53-mutant tumors Pre-incubation with a small-molecule p53 activator selectively induces cell arrest in normal cells, thereby protecting them from subsequent exposure to a classic S or M phase-specific cytotoxic drug without compromising the anticancer efficacy of the treatment.

For cyclotherapy to have the desired effects, i.e., shielding normal cells without diminishing the anticancer efficacy of the treatment, the protectant−therapeutic combination has to be chosen with great care. Cell cycle arrest does not guarantee protection from every cytotoxic agent. For instance, arresting cells in S-phase prior to exposure to the nucleotide analogue gemcitabine (*Gemzar*®) is likely to lead to synergistic cell killing rather than protection. Moreover, chemoprotection is unlikely to be effective if the anticancer drug outlives the cytostatic effect in cells. This is the case with classic DNA crosslinkers and intercalators, whose deleterious effects become apparent when cells re-enter the cell cycle. Preclinical p53-based cyclotherapy studies using cisplatin (*Platinol*®) and doxorubin (*Adriamycin*®) as second drug were indeed unsuccessful.

In a recent publication [10], we have investigated a series of cyclotherapy regimes involving four small-molecule p53 activators, tenovin-6 [11], leptomycin B (LMB) [12], nutlin-3 [13] and low doses of dactinomycin (LDactD) [14]. On normal cells in culture, these compounds have a reversible cytostatic effect, leading to the accumulation of cells in G1 and G2, and efficiently shield them from the cytotoxicity and nuclear aberrations caused by clinically approved S and M phase-specific poisons [10]. The quality of the protection attained is highlighted in Figure 2, which shows normal cells exposed to the tubulin poison vinorelbine (*Navelbine*®) with or without tenovin-6 pre-incubation. Generally tenovin-6 and LMB safeguard normal cells better than nutlin-3 or LDactD. However, from our systematic analysis [10], nutlin-3 emerged as the most promising chemoprotectant overall, showing good and highly-selective protection of normal cells from each anticancer drug tested, which is in agreement with previous data [8, 9, 15, 16].

**Figure 2:**
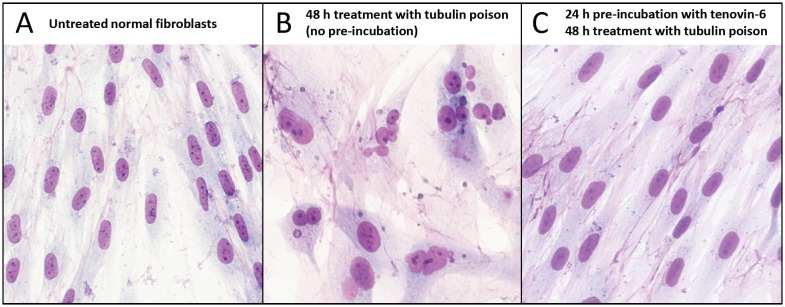
Effective chemoprotection of normal cells from nuclear aberrations caused by a tubulin poison Human normal dermal fibroblasts were (A) left untreated, (B) treated with 40 nM vinorelbine as a sole agent for 48 h or (C) pre-incubated with 3 μM tenovin-6 prior to exposure to vinorelbine. Cells were then left to recover for several days in fresh medium and stained with Giemsa.

Translating the concept of cyclotherapy into a clinical reality will involve a number of major challenges, including the following:

1. Further research is needed to identify suitable compounds to be used as chemoprotectants. Ideal candidates ought to elicit a reversible cytostatic response in normal cells over a wide range of concentrations. LMB is a very attractive compound from this point of view, since it is effective between 0.2 and 400 nM in cell culture [10]. Also, selectivity is critical and chemoprotectants should not induce cell cycle arrest in tumor cells. Our observations suggest that LDactD, for example, has a relatively narrow therapeutic window. For both normal and p53-mutant cancer cells, dactinomycin is cytotoxic at doses beyond 4 nM and mildly cytostatic below 2 nM [10, 17].
2. The cyclotherapy principle needs to be further validated *in vivo*. To date, a sole publication has reported that nutlin-3 can efficiently prevent neutropenia in mice exposed to an anticancer agent [16].
3. Cell culture studies strongly suggest that optimal cyclotherapy regimes will vary between patients. Therefore, recent advances in the development of freely available databases profiling the drug sensitivity of large panels of cancer cell lines and high-throughput technologies for screening patient samples are of great interest [18-21]. These constitute substantial progress towards the ultimate goal of individually tailored therapies.
4. The clinical approval of potential chemoprotectants constitutes a major time-limiting step. Tenovin-6 is a novel compound still under preclinical investigation [11], while a nutlin-like compound is currently undergoing phase 1 clinical trials. LMB's progress as a possible therapeutic is hindered by early reports of high toxicity *in vivo* as well as by this natural compound's lack of a ‘composition of matter’ patent. The future does, however, look brighter for novel LMB analogues [22]. Finally, an appealing alternative is to exploit drugs that are already in the clinic, such as dactinomycin, as chemoprotectants.

Since it is so frequently mutated in human cancers, the p53 tumour suppressor constitutes a highly attractive target for selective chemoprotection [23-25]. However, it is important to note here that other pathways can also be potentially exploited to selectively induce cell cycle arrest in normal tissue (cyclotherapy) or to lower its sensitivity via other chemoprotection strategies [26-31]. Apontes *et al*., [26], for example, explored the use of the immunosuppressant rapamycin (inhibitor of mTOR signalling) and the anti-diabetic drug metformin (activator of AMP kinase) as chemoprotectants. Furthermore, Raffaghello *et al*., [29] reported that short-term serum deprivation selectively protects normal cells in culture from the cytotoxicity of cyclophosphamide (*Endoxan*®) and improves the survival of mice exposed to high doses of etoposide (*Eposin*®), and preliminary dietary intervention studies in patients revealed a decrease in a range of side effects with fasting [32]. The differential effect of starvation on normal and cancer cells is mediated, at least in part, by insulin-like growth factor I (IGF-I) [33]. In another recent publication, Pabla *et al*., [28, 34] identified protein kinase C δ (PKCδ) as a key player in the nephrotoxicity and kidney damage associated with cisplatin-based cancer therapy and effectively exploited PKCδ inhibition to reduce cisplatin-induced apoptosis in renal proximal tubular cells in mice.

In summary, cyclotherapy could improve treatment outcomes by making it possible to escalate the dose or intensity of cancer therapy without aggravating toxicity in healthy tissue. Even if an increase in survival cannot be achieved in this manner, cyclotherapy could still prove to be an invaluable strategy to improve the quality of life of cancer patients. That is, together with other advances in chemo- and radio-therapy, cyclotherapy has the potential to open a therapeutic window for cancer treatment.
